# Microbial Efflux Pump Inhibitors: A Journey around Quinoline and Indole Derivatives

**DOI:** 10.3390/molecules26226996

**Published:** 2021-11-19

**Authors:** Giada Cernicchi, Tommaso Felicetti, Stefano Sabatini

**Affiliations:** Department of Pharmaceutical Sciences, University of Perugia, Via del Liceo, 06123 Perugia, Italy; giada.cernicchi@outlook.it (G.C.); stefano.sabatini@unipg.it (S.S.)

**Keywords:** efflux pump inhibitors, antibiotic resistance breakers, microbial efflux pumps, antimicrobial resistance, antibiotic resistance

## Abstract

Antimicrobial resistance (AMR) is a complex threat to human health and, to date, it represents a hot topic in drug discovery. The use of non-antibiotic molecules to block resistance mechanisms is a powerful alternative to the identification of new antibiotics. Bacterial efflux pumps exert the early step of AMR development, allowing the bacteria to grow in presence of sub-inhibitory drug concentration and develop more specific resistance mechanisms. Thus, efflux pump inhibitors (EPIs) offer a great opportunity to fight AMR, potentially restoring antibiotic activity. Based on our experience in designing and synthesizing novel EPIs, herein, we retrieved information around quinoline and indole derivatives reported in literature on this topic. Thus, our aim was to collect all data around these promising classes of EPIs in order to delineate a comprehensive structure–activity relationship (SAR) around each core for different microbes. With this review article, we aim to help future research in the field in the discovery of new microbial EPIs with improved activity and a better safety profile.

## 1. Introduction

The extensive use of antibiotics to treat microbial infections in humans and animals is paving the way to the development of resistant microorganisms. To date, antimicrobial resistance (AMR) represents a complex global public health issue with high costs for the healthcare sector, and the wider societal impact still largely unknown [1]. As a result of multidrug-resistant microbial infections, about 700,000 people worldwide die each year [2], 33,000 of which die in Europe, burdening the European Union with costs of EUR 1.5 billion annually [3]. Similarly, in the United States, more than 2 million people are infected with antibiotic-resistant microorganisms each year, with 35,000 deaths and an yearly cost to the health system estimated at USD 21 to USD 34 billion, coupled with more than 8 million additional days in hospitalizations [1]. 

To face AMR, besides discovering novel antibacterial agents, an alternative/parallel strategy relies on the approach of targeting microbial mechanisms underlying resistance. The idea of freezing resistance would allow antibiotics, which generated the resistance, to work again, thereby renewing our armamentarium to fight microbial infections. Over the years, molecules targeting factors involved in AMR have been named in different ways: adjuvant molecules, helper compounds or antimicrobial resistance breakers (ARBs) [4,5,6,7]. All of them share the lack of any antimicrobial activity of their own and the ability to synergize by different mechanisms with known antimicrobials, thus restoring the lost activity against resistant strains. Since microorganisms evolve resistance only for compounds exerting bactericidal or bacteriostatic effects, the lack of the antimicrobial activity in ARBs appears to be a strength for their potential use [8]. In addition, the combination of an ARB with an antibiotic could likely reduce the doses of the latter, thereby mitigating its side effects. Consequently, the use of ARB appears advantageous, even considering the great number of microbial proteins and factors not suitable for developing direct antibacterials, which could be exploited as promising targets [4]. In this regard, ARBs might also be considered as anti-virulence compounds when the mechanisms involved in AMR are associated with increased microbial virulence, thus suggesting their use as a mono-therapy [9]. The research for anti-virulence compounds exerting a poor selective pressure on microorganisms and potentially respecting gut microbiota is in its infancy, more so than the strategy to combine an ARB with an antibiotic, but this approach deserves much more attention [10].

However, the use of an ARB to fight AMR is challenging, owing to various potential issues, including the identification of correct biological assays demonstrating the effective ARB activity. Indeed, it is essential that the synergism between the ARB and the antibiotic relies on the expected mechanism of the ARB. Moreover, the candidate ARB molecule should overcome all the “journey” of a drug development and next potential drug–drug interactions or side effects when co-administrated with different antibiotics. However, the feasibility of this strategy can be sought in the success of the β-lactamase inhibitors [11], progenitor of the whole ARB class and currently deemed essential in the antibiotic therapy based on β-lactam antibiotics. 

Considering their peculiar role in AMR development, efflux pumps (EPs) represent a valuable target for developing ARB agents. EPs are typical trans-membrane proteins present both in prokaryotic and eukaryotic cells. They require an energy source (i.e., proton motive force) to extrude noxious compounds, such as substances synthetized by host organisms and a large array of antimicrobial agents [12]. Based on the amino acid sequence similarities, predicted secondary protein structures and phylogenetic relationship, microbial EPs are divided into six different families: ATP-binding cassette (ABC) superfamily, major facilitator superfamily (MFS), multidrug and toxic compound extrusion (MATE) superfamily, small multidrug resistance (SMR) superfamily, proteobacterial antimicrobial compound efflux (PACE) family, resistance–nodulation–cell Division (RND) superfamily [13]. In contrast, when classified according to the energy source used to extrude substrates, EPs fall in two classes; the former utilizes the hydrolysis of ATP (ABC superfamily) while the second class is an electrochemical gradient of sodium ions or protons (all the other families) [14].

EP inhibitors (EPIs) are to be considered a class of ARBs that may hold a pivotal role in fighting AMR. Since they are expressed in all microorganisms at a basal level, EPs are involved in several pathways functional to the microbial life [15]. However, during antibiotic treatment, the basal presence of EPs contributes to slightly decreased antimicrobial concentrations inside the microbial cells to sub-inhibitory levels, in turn producing an increase in the mutational transformations in microorganisms and promoting the development of target-based resistance [16,17,18,19,20]. Moreover, through the use of EPIs, the role of EPs in biofilm formation has been indirectly shown, by observing that pathogens overexpressing EP genes display a thicker biofilm than corresponding wild-type and EP genes knock-out strains. Although the mechanisms by which EPs contribute to the formation of biofilm are still unclear, the possibility that an EPI could inhibit biofilm is very intriguing [21,22,23].

Therefore, an EPI potentially appears effective: (i) in reducing insurgence of resistance in wild-type strains, (ii) in overcoming efflux-mediated mechanisms in resistant strains and (iii) in hindering biofilm production. 

However, the downside underlying the development of an ARB, such as an EPI, relies on the poor availability of quick and smart biological screenings able to identify active molecules early. In addition, in literature, molecules have often been described as EPIs when they are not owing to the poor knowledge of the efflux mechanisms and the lack of biophysical experiments validating a true EP inhibition. Thus, the presence of “fake” EPIs in literature is (i) facilitated by the poor experience in this field due to its novelty and (ii) encouraged by the exhausting research of molecules fighting AMR and by the fascinating approach underlying the identification of ARBs.

Among the hundreds of papers reported in literature on this topic, we noticed the presence of two scaffolds which were most employed to design and synthesize new synthetic EPIs. Accordingly, based on our experience in the search for EPIs, we collected information about the derivatives reported in literature as microbial EPIs and characterized by these two scaffolds (quinoline and indole) in order to highlight the potential of these classes for the development of potent EPIs. For a comprehensive description of all microbial EPIs present in literature, we suggest some recent review articles [10,24,25]. Indeed, herein, we focused the attention on the delineation of a clear structure–activity relationship (SAR) around quinoline and indole derivatives as microbial EPIs. Since an EPI should not possess any antibacterial activity on its own, all described derivatives are to be considered as not possessing this activity, unless indicated. Great attention has been given to the experiments proving EPI activity and to the evaluation of the toxic profile. In order to compare EPI activities, when possible, the synergistic activity of EPIs with antibiotics has been furnished as minimal potentiating concentration (MPC) able to reduce x-fold the antibiotic minimal inhibitory concentration (MIC) (MPCx). Below, EPI derivatives have been classified on the basis of the microorganisms where they are tested. In Table 1, all the microorganisms used to assess EPI activity for the cited compounds are reported. In addition, in Table S1, chemical names and structures of all described EPIs are reported.

## 2. *Staphylococcus aureus* Efflux Pump Inhibitors

*S. aureus* is a Gram-positive bacterium belonging to the family of Staphylococcacee. Grouped among the ESKAPE pathogens [44], *S. aureus* and especially its methicillin resistant strain (MRSA) are a great problem for the human health. It possesses several EPs belonging to different families, and some of them are able to extrude a wide array of compounds including many antibacterials commonly used in therapy and antiseptics. Indeed, the impact of EPs in the development of resistance in *S. aureus* is widely recognized, especially when considering their large overexpression in MRSA strains [45,46]. Among all the *S. aureus* EPs, NorA and MepA are the mostly studied and mainly involved in fluoroquinolone resistance. NorA consists of 388 amino acid residues and, as an MFS member, it is organized in 12 TM α-helices [47,48]. To date, no crystal structures are available, and little is known about the mechanism of efflux apart from how it functions by using the proton motive force. Over the years, many NorA EPIs have been discovered by three different approaches: (i) by screening libraries of natural or synthetic molecules; (ii) by repurposing molecules with known biological activity and (iii) by designing and synthesizing new compounds. The lack of an NorA crystal structure has strongly hampered the identification of potent NorA EPIs with no examples of structure-based drug design reported for EPI identification, which, therefore, relies on ligand-based drug design approaches or classical medicinal chemistry strategies. However, some efforts have also been directed towards the development of homology models coupled with computational studies to (i) perform virtual screening of potential NorA EPIs, (ii) propose the binding mode of some molecules inside the NorA EP, and (iii) understand how NorA could extrude its substrates [49,50,51].

### 2.1. Indole Derivatives

In 1999, the pioneering group of Neyfakh [52] performed a screening on 9,600 structurally diverse compounds with molecular weights ranging from 200 to 700 against a modified strain (ΔΔNA) of *B. subtilis* expressing the *norA* gene from a plasmid [53].

**Figure 1:**
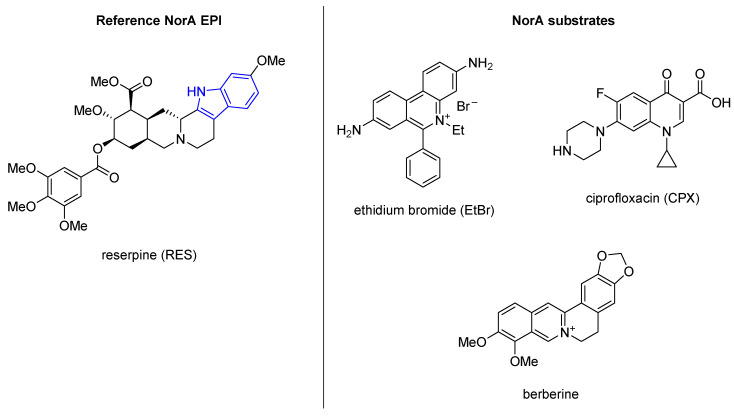
Reference NorA EPI reserpine (**RES**) and NorA substrates ethidium bromide (**EtBr**), ciprofloxacin (**CPX**) and berberine. Blue color is used to highlight the indole portion present in reserpine.

ΔΔNA strain derives from the previously built *B. subtilis* ΔΔ strain, where genes encoding the two main EPs Bmr and Blt have been inactivated [54]. By this modification, ΔΔNA strain exhibited a 20-fold increase in resistance to ethidium bromide (**EtBr**—Figure 1) with respect to its parent ΔΔ strain. 

Each compound was tested at the concentration of 20 µg/mL in combination with **EtBr** at 10 µg/mL, a concentration four-fold lower than its MIC [52]. Since the only known resistance mechanism for **EtBr** is its pump-mediated extrusion, the authors quickly identified which derivatives exhibited NorA EPI activity. In addition, specific NorA inhibition was assured because genes encoding for other EPs were silenced. Out of 9600 derivatives, 399 showed a four-fold **EtBr** MIC reduction while not having any bactericidal effect at the used concentrations. When checkerboard assays were performed with ciprofloxacin (**CPX**—Figure 1), 28 compounds retained activity up to 5 µg/mL, such as the reference compound reserpine (**RES**—Figure 1), while 11 of the 399 derivatives showed a higher EPI activity than **RES**, as they were still effective at 2.5 µg/mL.

**Figure 2:**
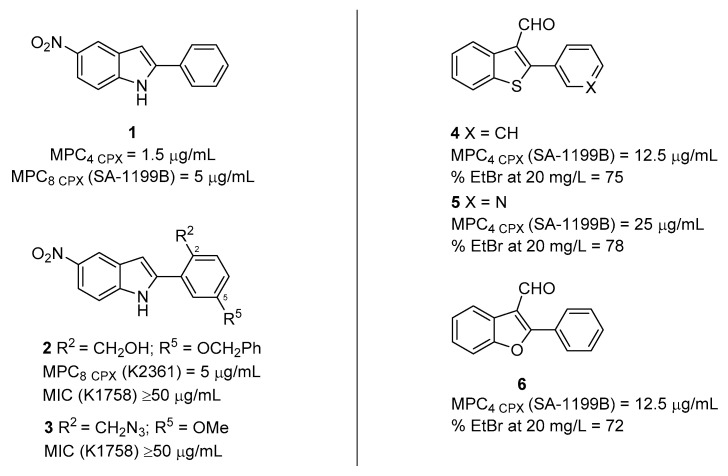
From indoles to benzothiophene and benzofuran EPIs.

Interestingly, among the 399 active compounds, 30 possessed an indole ring such as **RES,** and 11 derivatives were endowed with a biphenyl urea moiety. After checkerboard assays, in combination with **CPX** against SA-1199B, indole derivative **1** (Figure 2) resulted in one of the best compounds endowed with an MPC_4_ of 1.5 µg/mL. In addition, **1** was also able to prevent a fluorescence decrease in **EtBr** efflux assays on the ΔΔNA strain, similarly to **RES**. Unfortunately, the authors did not carry out a cytotoxicity evaluation on human cells, thereby foreclosing a potential use in in vivo models.

In 2005, Samorson et al. [55] explored the C-2’ and C-5’ positions of the phenyl ring on the C-2 position of the indole nucleus of derivative **1**, synthesizing a set of 10 derivatives. Compounds devoid of any antibacterial effect on their own (MIC ≥ 50 µg/mL) against three different *S. aureus* strains (K1758, 8325-4 and K2361—Table 1) were evaluated for their synergistic activity with berberine (a known NorA EP substrate—Figure 1). Compounds **2** and **3** (Figure 2), characterized by the presence on the C-2 phenyl ring of a primary alcohol or an azidomethyl group at the C-2’ position and of a benzyloxy or methoxy substituents at C-5’ position, respectively, emerged as the best derivatives able to potentiate berberine more than 15-fold (from >500 to 30 µg/mL) at a concentration of 0.8 and 1.5 µg/mL, respectively, against K2361 (Table 1). While a primary alcohol or an azidomethyl group were well tolerated at C-2’ coupled with a benzyloxy or methoxy substituents at C-5’, an acidic function at C-2’ resulted in the lack of berberine potentiation. On the other hand, regardless of substituent at C-5’ phenyl ring, a primary amine at C-2’ yielded derivatives with synergistic activity against *S. aureus* strains wild-type and *norA* deleted while lacking EPI activity against K2361 (Table 1). The best compound **2** was further evaluated, in comparison with **1**, for its ability to promote berberine accumulation and to synergize with **CPX** in the used *S. aureus* strains. Like **1**, derivative **2** showed a **CPX** MPC_8_ of 5 µg/mL and it was able, at 10 µg/mL, to promote berberine accumulation on K2361 strain (overexpressing *norA*—Table 1). However, a careful analysis of berberine accumulation curves showed that activity of compounds **1** and **2** could not be only due to NorA inhibition. Indeed, berberine accumulation in the three used strains only occurred in Δ*norA* strain K1758 (Table 1) producing values of relative fluorescence unit (RFU) of about 200 after 20 min, while no fluorescence was recorded in the wild-type *S. aureus* 8325-4 and *norA*++ K2361 strains (Table 1), indicating that berberine was completely extruded. When compounds **1** and **2** were tested at 10 µg/mL as the final concentration, RFUs drastically increased at values above 500 on all the used strains. Therefore, since the addition of compounds **1** and **2** caused such a high increase in the fluorescence in all strains (also those wild-type and Δ*norA*), the effect of berberine accumulation may be nonspecific for at least three different reasons. Indeed, compounds **1** and **2** could: (i) increase membrane permeability of *S. aureus* cells, thereby nonspecifically synergizing with berberine; (ii) also inhibit EPs other than NorA involved in berberine extrusion; (iii) emit fluorescence at the same wavelength as berberine. Unfortunately, no data about cytotoxicity against human cells was available. 

One year later, on the back of the good results obtained around the nitro-indole moiety as essential scaffold for NorA EPI activity, Fournier et al. [56] undertook an extensive synthetic effort to develop arylbenzo[*b*]thiophene and diarylthiophene derivatives with the aim to evaluate the importance of the indolic nitrogen atom for NorA inhibition. No compounds showed any antibacterial effect at concentrations lower than 100 µg/mL against *S. aureus* ATCC 25923 (Table 1); however, no MIC values were determined against SA-1199B (Table 1), the *S. aureus* strain used for synergistic assays, thus making it impossible to ascertain, though synergism was influenced by a direct antibacterial activity. A key requirement to possess synergistic activity in the arylbenzothiophene class was the presence of an aldehyde group at C-3 position coupled with substitutions on the C-2 phenyl ring in para position rather than meta or ortho positions. However, compounds with the aldehyde group at C-3 and an unsubstituted C-2 phenyl ring (**4**—Figure 2) or a C-2 pyridin-3-yl portion (**5**—Figure 2) showed promising MPC_4_ values (≤25 µg/mL). On the other hand, benzothiophene derivatives bearing –CN, a –OCH_3_, or a –OCH_2_CF_3_ groups at C-3 or even aryl moiety shifted from C-2 to C-3 exhibited MPC_4_ values ≥50 µg/mL. Interestingly, when the main nucleus was shrunk by benzene removal to give thiophene derivatives, the synergistic effect was completely lost, regardless of substituents. On the contrary, sulphur replacement of the benzothiophene with an oxygen atom yielded the benzofuran derivative (**6**—Figure 2) that retained the same activity as its close structural analogue **4**. For the best compounds **4-6**, NorA inhibition at 20 µg/mL was confirmed by **EtBr** efflux assays in comparison with **RES**. Finally, the cytotoxic profile was assessed for a large panel of derivatives against three different cell lines: keratin-forming tumor HeLa (KB), breast cancer (MCF7) and breast carcinoma (MCF7R). Unfortunately, derivatives **4** and **6**, at lower concentrations (10 µM) than those needed for NorA inhibition, showed a significant inhibition of the human cell growth. The only exception was derivative **5**, which exhibited a weak inhibition of KB cell growth and no significant effect against MCF7 and MCF7R; however, the tested concentration for cytotoxic assessment was about 10-fold lower than its MPC_4_. Thus, although the authors reported arylbenzo[*b*]thiophene derivatives as NorA inhibitors for the first time, much needs to be done to improve this chemical class to obtain more potent and safer compounds. Moreover, the authors should reconsider the initial assumption to remove the –NO_2_ group present in indole **1** in an attempt to increase NorA EPI activity. Indeed, a direct comparison of the EPI activity of **1** with the best benzothiophene derivative **4** highlights that indole moiety is preferred over the benzothiophene nucleus, even though the presence of the –NO_2_ group of **1** as well as the aldehyde of **4** could influence EPI activity, thereby hampering the comparison between the two nuclei.

**Figure 3:**
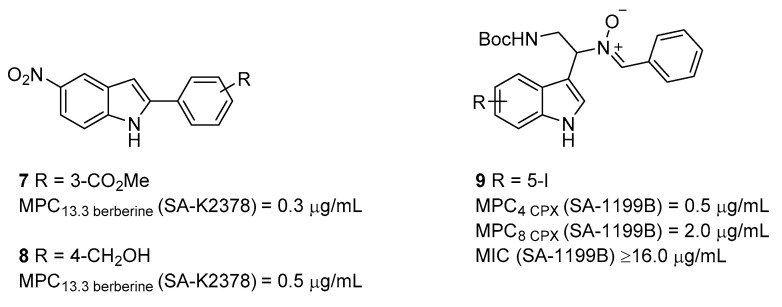
Indoles derivatives as NorA EPIs.

In 2008, as a continuation of the previous study [55], the group of Lewis [57] reported the synthesis and biological evaluation of 20 new 2-aryl-1*H*-indole derivatives with the aim to investigate the role of the –NO_2_ group in modulating the indole-mediated NorA inhibition. Thus, after the synthesis and evaluation of eight indole derivatives with different substituents at the C-5 position, the authors identified the –NO_2_ group as essential to possessing synergistic activity, in combination with berberine against the *S. aureus* wild-type (8325-4) and *norA* overexpressing (SA-K2378) strains (Table 1), with only the CN group that is potentially able to replace the NO_2_ group. Accordingly, the authors synthesized six new nitro-indole derivatives and their corresponding des-nitro compounds bearing different substituents on the C-2 aryl moiety. Synergistic activity, in combination with berberine, was evaluated as the MPC_13.3_ for each compound. As expected, all des-nitro indole derivatives showed poor synergistic activities, while nitro-indole analogues highlighted moderate to good effects. In particular, although less active than starting hit **1**, derivatives **7** and **8** (Figure 3) displayed an MPC_13.3_ of 1.0 µM (0.3 µg/mL) and 2.0 µM (0.5 µg/mL), respectively, against both *S. aureus* 8325-4 and SA-K2378 (Table 1). This comparable activity against the two used strains clearly highlights that both compounds have a nonspecific synergizing effect, as they reduce berberine MIC in the same manner against the wild-type and the *norA* overexpressing strains. In addition, although all compounds exhibited MIC values much higher than concentrations needed to reach the synergistic effect with berberine, the NorA inhibition was not proved by **EtBr** efflux assays for any of the compounds. No data about synergistic activity with **CPX** or other NorA antibacterial substrates was reported.

In 2014, Hequet et al. [58] further explored the role of the indole derivatives as NorA EPIs, focusing attention on the C-3 position. From a previous work aimed at discovering new indoles as antibacterial agents, the authors observed that the presence of an aldonitrone moiety at the C-3 position removed the antibacterial activity against different bacteria [59]. With the aim to improve solubility, they enlarged the indole series, further synthesizing 12 new indole analogues that were tested as NorA EPIs by evaluating their ability to inhibit, alone or in combination with **CPX**, the bacterial cells’ growth against SA-1199B strain (Table 1). By analyzing the synergistic activity of compounds at concentrations below their MIC values, it is possible to state that the presence of a halogen atom at C-5 position of the indole core is essential to displaying NorA EPI activity (best compound **9** MPC_4_ = 0.5 µg/mL, MPC_8_ = 2.0 µg/mL, MIC > 16.0 µg/mL—Figure 3). Indeed, derivatives, where the halogen atom was removed, showed a poor synergistic activity with **CPX** against SA-1199B (Table 1). This behavior is consistent with the importance of an electron-withdrawing group at the indole C-5 position, as also shown by the –NO_2_ group of derivative **1**. Furthermore, the indole derivative with halogen at C-6 retained significant synergistic activity (MPC_4_ = 4.0 µg/mL), strengthening the key role of the halogen on the indole core. On the other hand, aldonitrone moiety at C-3 was not essential to imparting NorA inhibition activity; indeed, when it was replaced with hydroxylamine or amide functions, synergistic activity with **CPX** against SA-1199B was not affected. However, in order to investigate whether the best derivatives specifically inhibited NorA function, the authors performed checkerboard assays for these compounds against SA-K2378 (*norA*+) (Table 1) and **EtBr** accumulation assays against SA-1199B (Table 1). All of them exhibited good synergistic activities with **CPX** and **EtBr** accumulation, thus confirming their mechanism of action as NorA EPIs. Cytotoxicity evaluation at 10 µM against three different human cell lines (human lung fibroblast—MCR5, human mouth carcinoma—KB and human colon tumor—HCT116) disclosed that the best compounds (including **9**) showed cytotoxic activity against human cells. 

**Figure 4:**
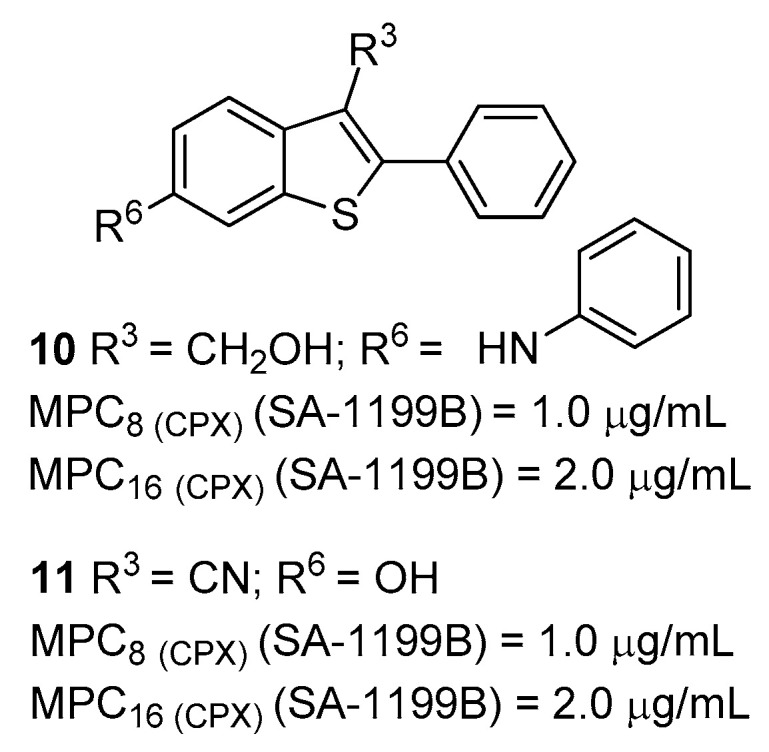
Benzothiophene derivatives as NorA EPIs.

Two years later, based on a previous work aimed at obtaining the 6-bromobenzo[*b*]thiophene-3-carbonitrile intermediate for Raloxifen synthesis [60], Liger et al. attempted to optimize the previously identified arylbenzo[*b*]thiophene derivatives (i.e., **4**) [61]. Although benzothiophene derivatives bearing a –CN group at C-3 did not show any NorA EPI activity in their previous work [56], the authors synthesized and tested a wide set of cyanobenzothiophene derivatives. However, the C-3 position was widely decorated with different electron-withdrawing groups in combination with an array of substituents, mainly at the C-6 position but also at C-4 and C-5. Once they excluded any antibacterial effect for all derivatives against ATCC 25923 and SA-1199B strains (Table 1), the authors assessed the synergistic effect of the compounds with **CPX** against SA-1199B. Although it was impossible to delineate a clear SAR around this class due to a lack of correlation between the activities of the combined substituents, some compounds showed promising synergistic activities at low concentrations. In particular, compounds **10** and **11** (Figure 4) exhibited an MPC_8_ of 1 µg/mL and an MPC_16_ of 2 µg/mL while showing an MIC > 128 µg/mL on tested strains. For both compounds, NorA inhibition was confirmed by **EtBr** accumulation assays at 20 µg/mL on SA-1199B by using **RES** as positive control. However, no synergistic assays against *S. aureus* strains wild-type or lacking NorA pump were performed, thereby not fully demonstrating the absence of a possible secondary synergistic mechanism, such as the increase in membrane permeability. Moreover, no human cell toxicity was assessed.

**Figure 5:**
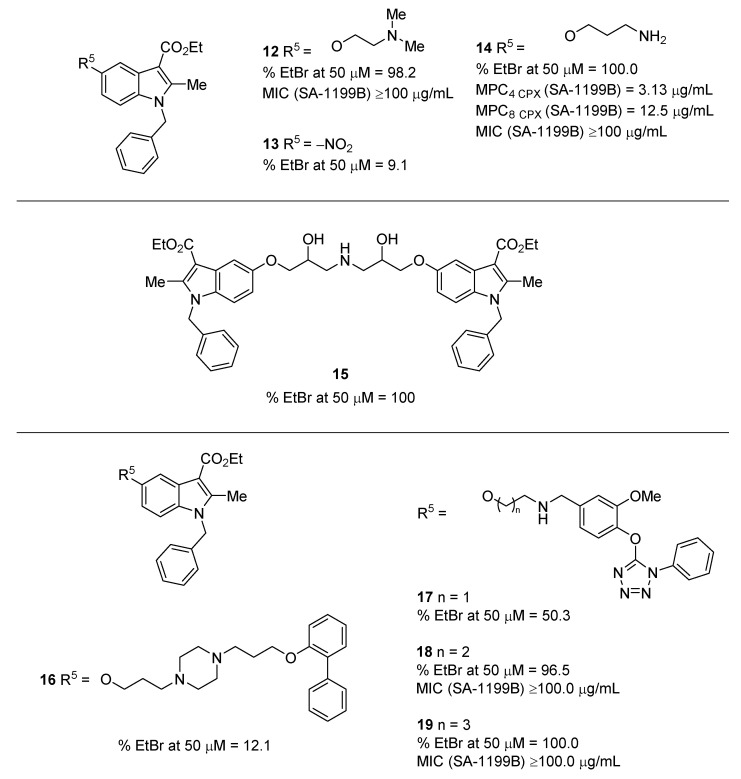
*N*-1 substituted indole derivatives as NorA EPIs.

In 2016, based on the literature data reported over the years around the indole core, Lepri et al. planned a rational design aimed at obtaining potent indole-based NorA EPIs [62]. The authors performed a wide effort to enrich SAR information around the indole core using a four-step design protocol, especially focusing attention on the *N*-1, C-3 and C-5 positions. The first substantial modification entailed the shift of the aryl from C-2 position, as the most potent indole EPIs **1**-**3** (Figure 2), to *N*-1 position of the indole core after demonstrating a similar spatial occupancy by superimposing the new *N*-benzyl indole with the 2-phenyl indole using FLAP software [63]. Thus, a set of benzyl-indole derivatives with an ethylic ester function at C-3 was synthesized with different substituents at the C-5 position in order to verify the role of the –NO_2_ group (present in **1**). All derivatives showed poor **EtBr** efflux inhibition at 50 µM against SA-1199B, with the exception of **12** bearing at C-5 a diethylamino ether chain (Figure 5). Interestingly, the indole derivative **13**, the direct analogue of **1** within this series of compounds and with the -NO_2_ group at C-5, was found to be inactive, thereby highlighting that the shift of the aryl moiety from C-2 to *N*-1 was detrimental (Figure 5). Although derivative **12** was the first indole NorA EPI derivative with an electron-donating group at C-5, the effect did not appear to be due to the electron-donating property of the C-5 substituent since other derivatives did not show any NorA EPI activity. On the contrary, the NorA EPI activity of derivative **12** would appear to be related to the presence of the protonable alkylamino chain, which was previously identified in the quinoline scaffold (see below) and is essential to imparting NorA inhibition [64]. Thus, the authors focused their attention on the substituent at C-5, introducing several different *O*-alkylamino chains. Overall, all 22 derivatives retained an excellent **EtBr** efflux inhibition, proving the key role of the protonable alkylamino chains in obtaining NorA inhibition.

Further modifications showed that the removal of the benzyl moiety from *N*-1 position led to a completely inactive derivative, thereby highlighting the importance of a lipophilic portion in this position. In addition, the removal or the hydrolysis of the ester at C-3 led to an increase in the antibacterial activity or the lack of **EtBr** efflux inhibition, respectively. Substitutions on the benzyl moiety gave few differences in terms of **EtBr** efflux inhibition regardless of the position of the substituent and its chemical property. Before selecting the best derivatives to evaluate their synergistic activity with **CPX** against SA-1199B (Table 1), the authors performed ADME studies on the best compounds overall, observing a moderate to good human metabolic stability after 30 minutes. However, the authors selected only some compounds to be evaluated for their synergistic activity, with **CPX** against SA-1199B and the wild-type SA-1199 (Table 1). The three derivatives (**14** included as representative) (Figure 5) showed an MPC_4_ of 3.13 µg/mL and an MPC_8_ of 12.5 µg/mL against SA-1199B while not showing any significant synergistic effect against the wild-type SA-1199. 

In conclusion, the authors performed a wide effort to enrich SAR around the indole core and identified new indole derivatives with good NorA inhibition, even without the –NO_2_ group at C-5. The best derivatives showed synergistic activity comparable to the starting hit **1** but, unfortunately, the authors did not deepen the studies around these compounds to confirm if their synergistic activity was due to the NorA inhibition by checkerboard assays against *norA* knock-out and *norA* overexpressing *S. aureus* strains. Nevertheless, the wide SAR work has strengthened the information in a view to obtain more potent indole-based NorA EPIs.

One year later, starting from the good **EtBr** efflux inhibition activity of the side product **15** (Figure 5) obtained during the synthesis of some derivatives in the previous work [62], the same authors planned a rational design aimed at performing a scaffold-hopping approach to obtain unsymmetrical derivatives of **15** [65]. Thus, through a virtual screening on the SPECS database using FLAP, authors identified two fragments to replace the symmetric portion of **15** in order to synthesize derivatives **16** and **17** (Figure 5), which unfortunately showed poor **EtBr** efflux inhibition. Thus, after a chemical optimization, derivatives **18** and **19** (Figure 5) showed an excellent **EtBr** efflux inhibition (low IC_50s_) coupled with high MIC values. Synergistic activity with **CPX** against SA-1199B was reported with FIC values, thus making difficult to obtain the exact MPC value for each compound. In addition, no synergistic evaluation was performed against specific *norA* knock-out and overexpressing strains as also in the previous work [62].

**Figure 6:**
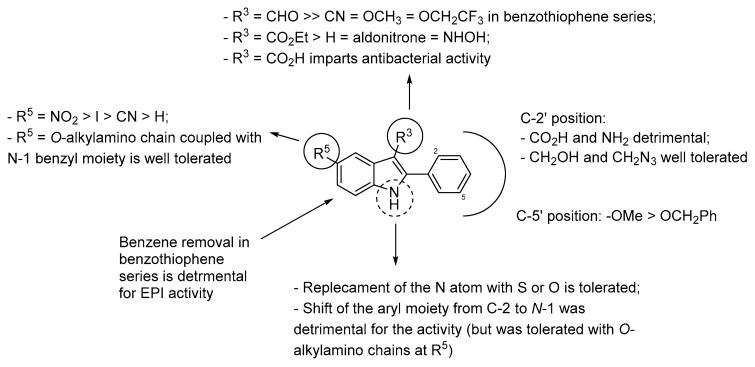
SAR of indole derivatives as NorA EPIs.

Taking into account all the indole analogues reported over the years as NorA EPIs, we tried to build a comprehensive SAR, as depicted in Figure 6. Since many groups focused their efforts on the identification of indole derivatives as NorA EPIs, SAR information is often related to the series of each research group, making it difficult to rationalize clear guidelines to design potent NorA EPIs. However, it is interesting to note that the indole core can be also replaced by benzothiophene or benzofuran moieties if properly substituted. On the other hand, regarding the indole core, the presence of a NO_2_ group at the C-5 position appears essential when a phenyl moiety is at C-2, while *O*-alkylamino chains at C-5 yielded potent derivatives when coupled with the aromatic portion shifted to the *N*-1 position. Of note, a carboxylic function was always poorly tolerated, affording derivatives lacking NorA EPI activity.

### 2.2. Quinoline Derivatives

**Figure 7:**
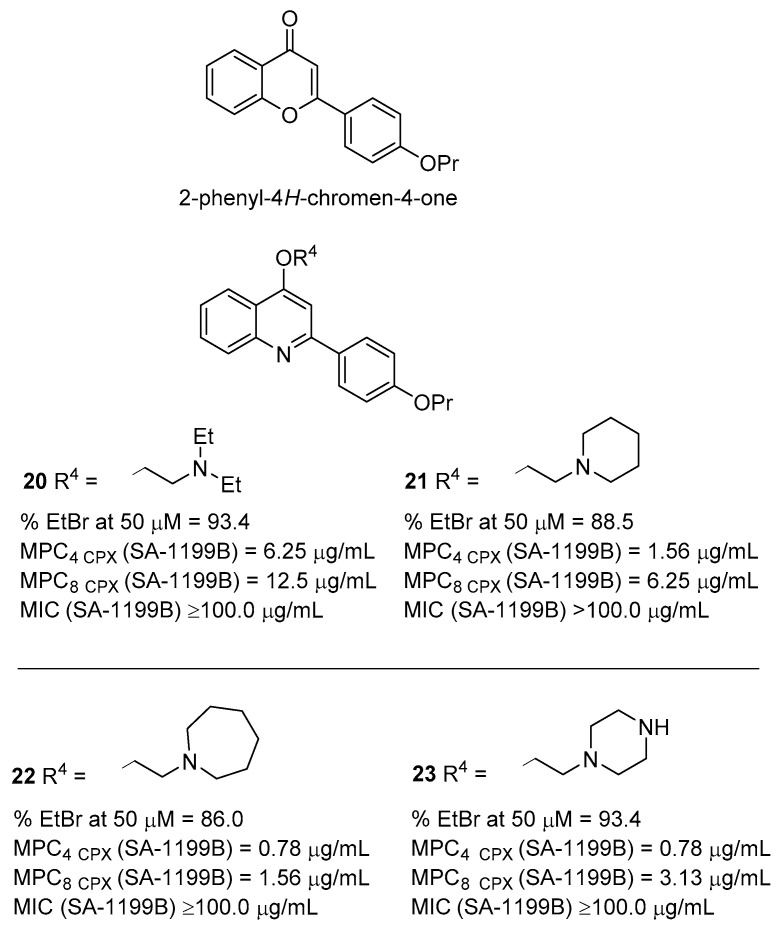
From flavone to quinoline nucleus.

Inspired from the 2-phenyl-4*H*-chromen-4-one, a common scaffold of the flavone and flavolignane EPIs [66], in 2011, Sabatini et al. started their work around the quinoline derivatives as potent NorA EPIs [67]. Initially, by replacing the endocyclic oxygen of the chromene core with a nitrogen atom, the 2-phenylquinolone core was obtained and, subsequently, decorated with different polar chains based on the promising results of some MDR inhibitors [68]. During the synthesis of the *N*-alkylated quinolones, *O*-alkylated 2-phenylquinoline derivatives were also obtained, proving to be the best NorA EPIs of the series. Indeed, derivatives **20** and **21** (Figure 7), devoid of any antibacterial activity on their own, showed the best results in terms of both **EtBr** efflux inhibition and synergistic activity with **CPX** against SA-1199B (MPC_4_ of 6.25 and 1.56 µg/mL, respectively). Therefore, an initial SAR around the quinoline core was delineated with alkylamino chains boosting the NorA EPI activity when present on the oxygen at the C-4 position (i.e., derivatives **20** and **21**), rather than at the *N*-1 position or on the oxygen at the C-4’ position of the C-2 phenyl ring. In parallel, the best substituent for the C-4’ position of the C-2 phenyl ring was a –OPr group, which was preferred over a free –OH or other alkyl ethers such as –OMe or –OEt. Noteworthily, derivatives **20** and **21** also showed good synergistic activity with **CPX** against SA-K2378 (Table 1) while no significant effect was observed against the *S. aureus* strains where NorA was poorly or not present (SA-1199 and SA-K1902—Table 1). 

Further optimization focused on the oxygen at the C-4 position of the 2-phenylquinoline core led to the design and synthesis of a wide set of analogues with different *O*-alkylamino chains [64]. When tested for evaluating their **EtBr** efflux inhibition and synergism with **CPX** against SA-1199B (Table 1), most of the compounds showed excellent results. In particular, derivatives **22** and **23** (Figure 7) showed MIC values ≥ 100 µg/mL, an MPC_4_ of 0.78 µg/mL and an **EtBr** efflux inhibition of about 90% at 50 µM. In addition, both compounds exhibited excellent synergistic activity with **CPX** against SA-K2378 (Table 1) and no effect against the other *S. aureus* strains with poor or no NorA expression (SA-1199 and SA-K1902, respectively—Table 1). 

**Figure 8:**
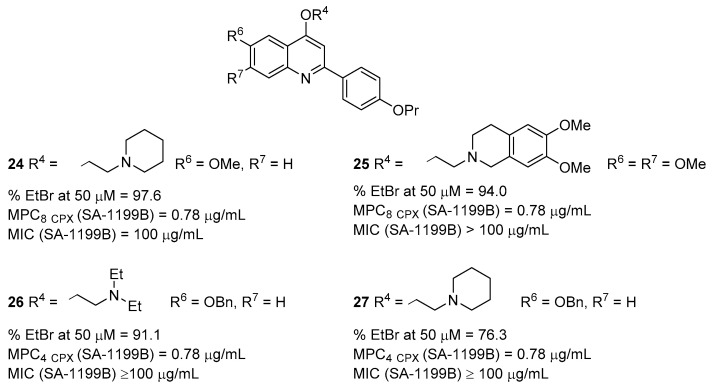
C6-modified *O*-alkylamino quinolines **24**–**27** as NorA EPIs.

Once it was reported that *O*-alkylamino chains at C-4 position were needed to impart NorA EPI activity to quinoline derivatives, further efforts were directed towards the functionalization of the “naked” benzene ring of the quinoline core [69]. Based on the large presence of the -OMe group on different known NorA EPIs, the functionalization of the quinoline C-5, C-6, C-7 and C-8 positions with –OMe groups was planned, maintaining *p*-OPr group at C-2 phenyl ring and selecting the best *O*-alkylamino chains at C-4. Biological assays showed that the –OMe introduction on the quinoline core yielded more potent NorA EPIs than des-methoxy analogues. In particular, the introduction of one –OMe at C-6 or two –OMe groups at the C-6 and C-7 positions resulted the best combinations, with compounds **24** and **25** (Figure 8) that emerged as the best derivatives, showing an **EtBr** efflux inhibition ≥ 90%, high MIC values (100 and >100 µg/mL, respectively) and an MCP_8_ of 0.78 µg/mL against SA-1199B (Table 1). In parallel, both compounds exhibited high synergism with **CPX** against SA-K2378 and no effect against SA-1199 and SA-K1902 (Table 1). Interestingly, compounds **24** and **25** also showed a non-toxic profile when tested on human liver epithelial (HepG2) cells, exhibiting CC_50_ values of 42.0 µg/mL for **24** and 74.0 µg/mL for **25**. As a confirmation, CC_50_ values > 100 µg/mL were observed against the human monocytic cell line (THP-1). Accordingly, both compounds possessed a CC_50_/MPC_8_ ratio > 128-fold. Interestingly, at 0.78 µg/mL, neither compounds modified the membrane proton motive force needed for NorA function, thus disclosing that NorA inhibition was not due to a nonspecific effect. In addition, both compounds also showed EPI activity towards MepA EP. Guided by the promising results obtained with the 6-OMe derivative **24**, further efforts were directed towards the investigation of the C-6 position of the quinoline core [70]. Considering the essential role of the alkylamino chain at the C-4 position of the quinoline, different analogues were designed with two *O*-alkyl-basic chains at C-4 and C-6 positions [70]. In addition, the intermediates C-6 *O*-benzyl and free OH analogues were used as counterparts to investigate the SAR. Although almost all the derivatives showed excellent inhibition of the **EtBr** efflux at 50 µM, only C-6 benzyloxy derivatives **26** and **27** (Figure 8) exhibited potent synergism with **CPX** against SA-1199B (Table 1). Indeed, both of them showed an MPC_4_ of 0.78 µg/mL coupled with poor or no synergistic effect against the wild-type strains ATCC 25923 and SA-1199 (Table 1). Evaluation of the cytotoxicity on HepG2 cells disclosed a significant toxicity, with CC_50_ values of 12.26 µg/mL (for **26**) and 33.83 µg/mL (for **27**). However, the combination of CC_50_ values with MPC_4_ suggested a promising selectivity of action for both compounds.

**Figure 9:**
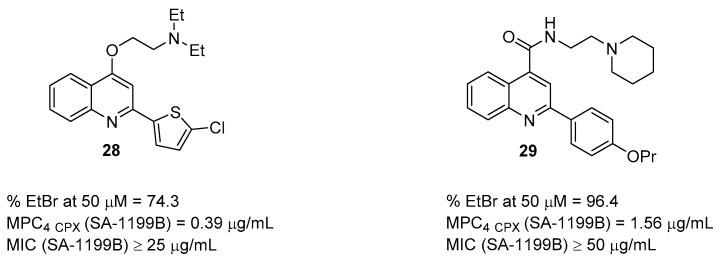
C-2 chlorothiophene-quinoline **28** and C-4 carboxy-quinoline **29** as NorA EPIs.

Two years later, the same authors focused their efforts on the exploration of the C-2 position of the quinoline core by replacing the 4’-propoxyphenyl substituent with differently substituted pyridine or thiophene moieties [71]. Starting from **20** (Figure 7), 16 new analogues were designed and synthesized by combining eight new C-2 aryl quinoline scaffolds with two different chains at C-4 (*O*-ethyl-*N*,*N*-diethylamino and *O*-ethylpiperidine). In checkerboard assays with **CPX**, chlorothiophene derivative **28** (Figure 9) showed the best synergistic effect, with an MPC_4_ of 0.39 µg/mL. By considering SAR information, the pyridine ring at the C-2 position was detrimental to NorA EPI activity, while thiophene moiety yielded some promising analogues, including the best compound **28**. NorA inhibition was confirmed on SA-1199B by: (i) **EtBr** efflux assays at 50 µM with **28** that showed an inhibition percentage ≥ 70% and (ii) membrane polarization assays with **28** depolarizing *S. aureus* membrane less than 20% when tested at 5 µg/mL. In addition, **28** exhibited a CC_50_ value of 6.33 µg/mL on THP-1 cells, showing a promising CC_50_/MPC_4_ ratio of 16.

Recently, with the aim to enrich the array of NorA EPIs, the same research group performed a scaffold-hopping approach of the quinoline core followed by a pharmacophore-based virtual screening [72,73]. Therefore, the quinoline-4-yloxy core of **20** (Figure 7) was replaced by several scaffolds arising from the smart fragmentation of approved drugs to generate a virtual library that was screened on two previously reported pharmacophore models for NorA EPIs [74]. Virtual hits with nine different scaffolds (quinoline-4-carboxamides, phthalazin-1(2*H*)-ones, benzimidazoles, pyridine, 1,7-naphthyridine, 1,8-naphthyridine, isoquinoline and quinazoline) were synthesized and evaluated as NorA EPIs. Although 2-arylquinazoline were found to be the best derivatives, two quinoline-4-carboxamide derivatives (compound **29** as representative) (Figure 9) showed promising synergism with **CPX** against SA-1199B with an MPC_4_ of 1.56 µg/mL. In addition, **EtBr** efflux inhibition on SA-1199B was of 96%, while synergistic activity with **CPX** was not observed against *S. aureus* strains lacking *norA* gene, thus confirming NorA inhibition as the potential mechanism of action. Cytotoxicity was also measured on two different cell lines (THP-1 and A549), which, in the presence of **29** at 3.13 µg/mL, exhibited a vitality of 62 and 100%, respectively. In addition, experiments also showed that isoquinoline and pyridine derivatives completely lost NorA EPI activity, thus suggesting important SAR information.

**Figure 10:**
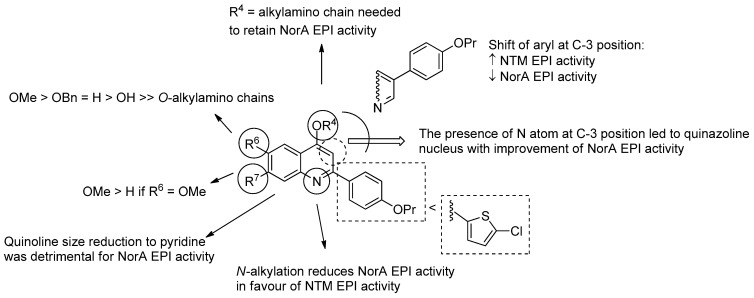
SAR of quinoline derivatives as NorA EPIs.

Considering the high number of reported articles from the same research group about quinoline derivatives as NorA EPIs, SAR around this core was more robust than for indole derivatives. Indeed, the picture in Figure 10 fully describes the state of the art around the chemical information to obtain potent quinoline analogues as potent NorA EPIs. Briefly, since size core reduction to pyridine yielded inactive derivatives, we can consider the quinoline core as essential, even though quinazoline nucleus gave potent derivatives. The shift of the C-2 aryl moiety to the C-3 position afforded derivatives less potent as NorA EPIs, while improving NTM EPI activity (see below), similarly to what happens for the position of the alkylamino chain if moved from the oxygen at C-4 to the *N*-1 position. 

In addition, at the C-6 position, the introduction of a free OH group or a second *O*-alkylamino chain was detrimental to NorA EPI activity, while the presence of a OMe group improved the activity and was preferred over a benzyloxy or a hydrogen. Interestingly, a OMe group at the C-7 position was well tolerated when a second OMe group was at C-6; indeed, mono-methoxy C-7 derivatives were less active than des-methoxy analogues.

## 3. Gram-Negative Efflux Pump Inhibitors

In 2017, the WHO published a list of antibiotic-resistant priority pathogens, and among these, the majority belongs to the Gram-negative bacteria class. These bacteria can cause serious diseases, such as pneumonia, blood-stream infections, meningitis and peritonitis, particularly in immune-compromised individuals. The nosocomial infections caused by Gram-negative pathogens are the harder issue for healthcare professionals due to the capability of microorganisms to acquire resistance, making pharmacological treatments ineffective. Unlike Gram-positive bacteria, Gram-negative bacteria have an outer membrane (OM), which is the main reason for resistance to a wide range of antibiotics, such as *β*-lactams, quinolones, tetracyclines [75]. The OM contains porins and other proteins that allow the passage of small molecules as well as hydrophilic antibiotics, while hydrophobic drugs can pass through a diffusion pathway [76]. Therefore, the alteration of hydrophobic properties coupled with structural mutations, including the overexpression of EPs, can contribute to the onset of resistance [77].

The most known EPs in Gram-negative bacteria are AcrB of *E. coli* and MexB of *P. aeruginosa*, belonging to RND family, whose crystal structures are available (PDB codes: 1IWG and 3W9I, respectively) [78,79]. Spreading within the Gram-negatives membranes, RND transporters form a tripartite assembly to give the AcrAB–TolC EP in the case of *E. coli* or MexAB–OprM of *P. aeruginosa* [80]. These transporters are much larger than MFS and are typically composed of approximately 1000 amino acid residues arranged in 12 TM α-helices. RND transporters are organized in trimers, and the substrate efflux is coupled with a proton movement [13]. In recent years, the research around RND transporters has experienced strong progress, thereby shedding light on the mechanisms involved in the efflux and opening the way for a rational identification of EPIs. Good progress has been made especially against *E. coli* and *P. aeruginosa*, where some pyranopyridines strongly potentiated the antibacterial activity of **CPX**, levofloxacin and piperacillin [81,82,83,84]. For one of these derivatives, the co-crystal structure (PDB code: 5ENO) [85] is also available while binding the AcrB subunit of AcrAB–TolC EP of *E. coli*. To date, for some pyranopyridines are ongoing in vivo studies [84]. Another interesting example is shown by a pyridopyrimidine derivative, whose co-crystal structures with AcrB and MexB are available (PDB codes: 3W9H and 3W9J, respectively) [79]. Unfortunately, the best pyridopyrimidine derivative exhibited toxic and pharmacodynamic issues in vivo [86].

### 3.1. Quinoline Derivatives

**Figure 11:**
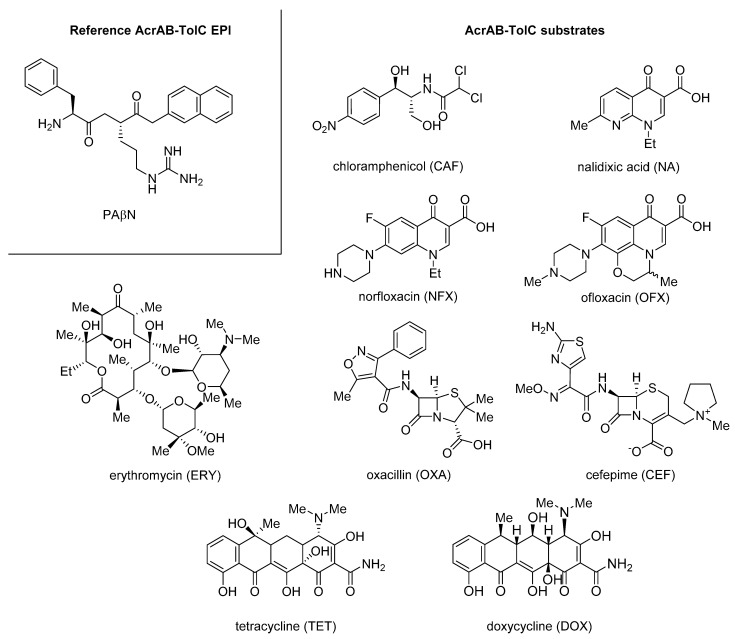
Reference AcrAB–TolC EPI **PAβN** and AcrAB–TolC substrates chloramphenicol (**CAF**), nalidixic acid (**NA**), norfloxacin (**NFX**), ofloxacin (**OFX**), oxacillin (**OXA**), cefepime (**CEF**), erythromycin (**ERY**), tetracycline (**TET**) and doxycycline (**DOX**).

**Figure 12:**
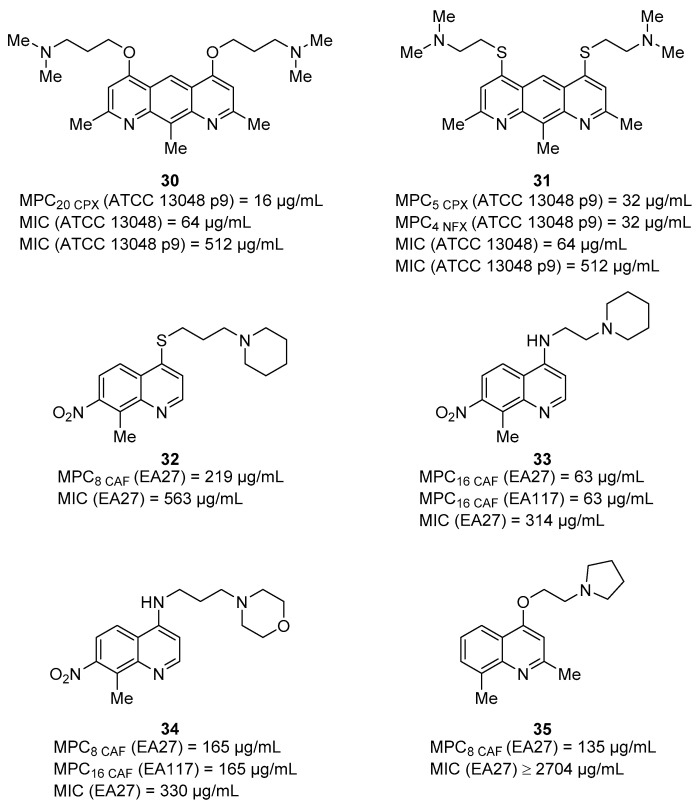
Quinoline derivatives (**30**–**35**) reported as Gram-negative EPIs.

One of the first examples regarding quinoline acting as Gram-negative EPIs reported in literature goes back to when Chevalier et al. [87], in 2001, described a series of pyridoquinoline analogues as inhibitors of different EPs in *Enterobacter aerogenes*. All derivatives were tested alone or in combination with **CPX** (Figure 1) and norfloxacin (**NFX**—Figure 11) against the wild-type strain ATCC 13048 and its parent, ATCC 13048 p9 (Table 1), harboring a plasmid overexpressing *marA* gene, an activator of EPs expression [88,89], which was found to be 20-fold more resistant to **CPX** (MIC moved from 0.025 to 0.5 µg/mL). Compounds showed modest MIC values against the wild-type strain (64 µg/mL) that significantly increased against the resistant strains (512–1024 µg/mL), likely resulting from EP substrates. When combined at 16 µg/mL with **CPX** or **NFX** against both strains, compounds **30** and **31** (Figure 12) exhibited a promising synergistic effect with **CPX** by reducing by 20- and 5-fold, respectively, with its MIC only against ATCC 13048 p9. Surprisingly, only **31** retained a synergistic effect with **NFX**. EPI activity was demonstrated by the accumulation of labeled **NFX** in ATCC 13048 p9.

In 2003, Gallo et al. reported a series of quinoline ethers or quinoline thio-ethers synergizing with **CAF** (Figure 11) against the resistant *E. aerogenes* strain (EA2—Table 1). Among all derivatives, compound **32** (Figure 12) exhibited the best activity, with an MPC_8_ of 500 µM (173 µg/mL) (MIC **CAF** moved from 512 to 64 µg/mL) [90].

In the same year, Mallea et al. reported a series of alkylaminoquinolines as inhibitors of the AcrAB–TolC EP in *E. aerogenes* [91]. Compounds **33** and **34** (Figure 12), with an MIC of 1 mM (314 and 330 µg/mL, respectively) on EA27, emerged as promising compounds by showing a chloramphenicol (**CAF**) MPC_16_ of 0.2 and 0.5 mM (63 and 165 µg/mL), respectively, against EA27 strain overexpressing the AcrAB efflux system (Table 1). SAR information highlighted the essential role of some alkylamino chains, such as piperidinoethyl and morpholinopropyl. Indeed, when diethylamino, dimethylamino or di-isopropylamino chains were linked to the aminoquinoline core, there was a significant reduction in the synergistic activity with **CAF [91]**. The best derivative **33** was also able to increase by the intracellular concentration of radiolabeled **CAF** in the EA27 strain 3-fold, similarly to PAβN, a known nonspecific Gram-negative EPI [92]. In addition, **33** was evaluated for its ability to decrease MIC values of structurally unrelated antibiotics (**CAF**, **NFX**, tetracycline (**TET**) and cefepime (**CEF**)—Figure 11) against different *E. aerogenes* strains in which decreasing porins levels were associated with the increasing of MDR. Data showed that compound **33** at 0.2 mM (63 µg/mL) mostly increased the susceptibilities to NFX (8-fold MIC reduction) and **CAF** (16-fold MIC reduction) when tested against EA27 and EA117 strains (Table 1), highlighting a better synergistic effect than PAβN. From these preliminary results, the class of alkylaminoquinoline seemed to be promising to develop Gram-negative EPIs, but further investigation should be made.

In 2004, Chevalier et al. described a series of quinolines, with two methyl groups at C-2 and C-8 positions and an ether functionality at C-4 bearing an alkylamino chain, as Gram-negative EPIs [93]. From an initial screening in combination with **CAF** against EA27, compound **35** (Figure 12—MIC > 10 mM) exhibited the best synergistic activity, with an MPC_8_ of 500 µM (135 µg/mL). The synergistic activity of **35** was not related to bacterial membrane disruption, as demonstrated by two different experiments on EA27 strain measuring: (i) potassium leakage and (ii) *β*-lactamase localization in the periplasm. In addition, it was reported that compound **35** at 1 mM was able to synergize with different antibacterials, such as **CAF**, **NFX** and **TET**, against different strains, such as EA3, synthesizing channel-altered porins, and EA117 and KP55 (*Klebsiella pneumoniae*), producing very small porin amounts. Interestingly, derivative **35** lost its synergistic activity with **CAF** and **NFX** when tested against two different modified EA27 strains where AcrA or TolC were deleted, thus suggesting a specific mechanism of action involving the inhibition of AcrAB–TolC EP. Regarding SAR information, it was evident that only the ethylpyrrolidine chain on the oxygen at the quinoline C-4 position gave the derivative **35** with EPI activity, while other alkylamino chains, though very similar like ethylpiperidine, afforded compounds without this activity. 

**Figure 13:**
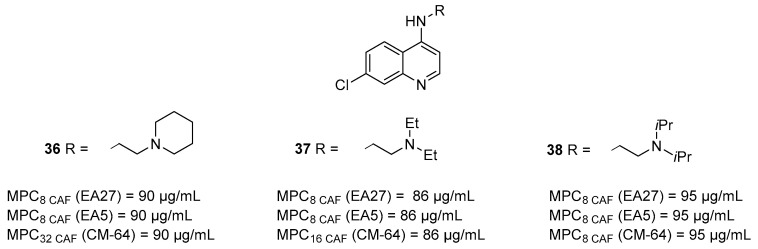
Quinoline derivatives (**36**–**38**) reported as Gram-negative EPIs.

Few years later, Ghisalberti et al. reported a series of chloroquinolines as potential EPIs towards antibiotic-resistant *E. aerogenes* isolates [94]. Among the six synthesized derivatives, decorated with different alkylamino chains at nitrogen atom at the C-4 position, three compounds (**36**–**38**—Figure 13) at 0.310 mM (86–95 µg/mL) showed a significant reduction in **CAF** MIC (by 8-fold) against EA27 and EA5 strains and by 32, 16 and 8-fold, respectively, against the CM-64 strain. In parallel, all three compounds did not show any antibacterial activity on their own and any synergistic effect when tested in combination with **CAF** against the wild-type *E. aerogenes* ATCC 13048. 

In 2016, Machado et al. reported an in-depth study on the role of a previously identified NorA EPI **23** (Figure 7) [64] with a 2-phenylquinoline scaffold as an *E. coli* EPI [95]. During the first step of the study, aimed at excluding any antibacterial activity of **23** against three different *E. coli* strains (the wild-type AG100, the AcrAB-deficient AG100A and AcrAB-overexpressing AG100_tet_—Table 1), a clear trend was evident. Indeed, **23** showed MIC values of 256 (AG100), 32 (AG100A) and >256 µg/mL (AG100_tet_), highlighting that the presence of AcrAB significantly influenced the antibacterial activity of **23** and suggesting that it could be considered an AcrAB substrate. However, the synergistic activity of **23** was evaluated in combination with ofloxacin (**OFX**), oxacillin (**OXA**), **TET** and **EtBr** against the three *E. coli* strains. The best results were obtained against AG100_tet_ by the combination of **23** 80 µM (31 µg/mL) with **OFX** (four-fold MIC reduction) and of **23** 40 µM (16 µg/mL) with **TET** (four-fold MIC reduction). In parallel, at the same concentration of **23**, no significant synergism with **OFX** and **TET** was appreciated against AG100 and AG100A strains, thus suggesting an EPI activity dependent on the overexpression of *acrAB*. No synergism was observed between **23** and **OXA** or **EtBr** against all three *E. coli* strains. The EPI activity of **23** was also confirmed through the real-time fluorometry using **EtBr** and determining the relative final fluorescence (RFF) values against AG100, AG100A and AG100_tet_ *E. coli* strains. The results were compared with values obtained for reference compounds (**PAβN** and **CPZ**); compound **23** showed an RFF of 13.1 against the wild-type strain, indicating a strong ability to interfere with **EtBr** efflux compared to **PAβN** and **CPZ,** which showed RFF values of 8.1 and 0.7, respectively. Considering the strain that overexpresses AcrAB–TolC (AG100_tet_), the quinoline derivative showed an RFF of 5.2, slightly less than **CPZ** but four times higher than PAβN, thus suggesting that **23** was able to promote the accumulation of **EtBr** in *E. coli* through the inhibition of the AcrAB. When the effect of **23** on the bacterial membrane potential was evaluated using the BacLight Bacterial Membrane Potential Kit, depolarized cells were over 85%; a value comparable to that was observed for the positive control CCCP (over 95%). Moreover, the possible effects of **23** on the membrane integrity of *E. coli* was evaluated at different concentrations, and no alteration of cell membrane integrity was detected after exposure at 80 µM (31 µg/mL ^1^/_8_ MIC) of **23**. Unfortunately, the promising activity of **23** was obtained at concentrations toxic for human monocyte-derived macrophages cells (CC_50_ = 11 µM/4.24 µg/mL). Information acquired from these preliminary studies displayed **23** as a promising template to develop *E. coli* EPIs, even though some drawbacks should be considered. Indeed, **23** likely was a AcrAB substrate and proved to interfere with PMF, thus indirectly causing the inhibition of the AcrAB–TolC efflux system. Further investigation should be done to define the SAR and improve the safety profile of the compound.

Regarding the SAR information acquired around all quinoline derivatives as Gram-negative EPIs, it was evident that the presence of an alkylamino chain was essential on the heteroatom at C-4 position on the quinoline core. However, unlike quinoline NorA EPIs, the delineated SAR was less clear, with no information about the best side chain to be introduced at the C-4 position. There is no preference for the heteroatom at the C-4 position needed to link the chain with the central scaffold. In addition, the presence of a NO_2_ group at the C-7 position as well as the methyl group at C-8 appear important for the EPI activity of some quinoline derivatives. However, this SAR information mostly came from the works of the research group of Jean-Marie Pagès, and most of them were not identified in the quinoline derivatives reported by Sabatini et al. that lacked both NO_2_ and Me groups, while having the phenyl ring at the C-2 position.

### 3.2. Indole Derivatives

**Figure 14:**
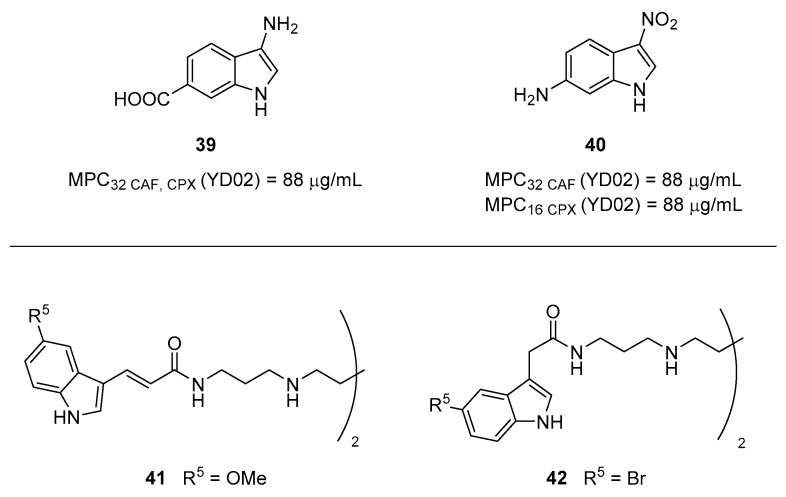
Indole derivatives reported as Gram-negative EPIs.

In 2010, Zeng et al. reported a series of indole derivatives as AcrAB–TolC EPIs able to synergize with different antibiotics such as **CAF**, **TET**, erythromycin (**ERY**) and **CPX** against different *E. coli* strains (including the wild-type ATCC 25922 and the resistant YD02—Table 1) [39]. By synergistic assays, compound **39** (Figure 14) at 0.5 mM (88 µg/mL) was able to reduce the **CAF** and **CPX** MICs by 32-fold against the YD02 strain. In parallel, compound **40** (Figure 14) at 0.5 mM (88.0 µg/mL) showed synergistic activities with all the used antibacterials, with MIC reductions ranging from 4 to 32-fold against the YD02 strain. Poor synergistic effects of **39** and **40** with antibacterials were observed against the wild-type strain ATCC 25922 (Table 1). Unfortunately, for the two best compounds, MIC evaluation, checkerboard assays and accumulation assays of antibiotics were not performed. 

In 2021, Cadelis et al. reported a series of indole-based spermine derivatives as Gram-negative antibiotic adjuvants [96]. An initial set of synthesized indole derivatives was evaluated for the ability to potentiate doxycycline (**DOX**) activity against the *P. aeruginosa* strain ATCC 27853 (Table 1). The evaluation of the antibiotic adjuvant activity of the best compounds (including **41** and **42**—Figure 14) was next extended towards different Gram-negative bacteria in combination with different antibiotics. In addition, derivative **42** at scalar concentrations was combined with three doses of four different antibiotics against four different bacteria. Results show a significant synergistic effect mainly when combined with (**DOX**) against *P. aeruginosa*, *E. coli* and *K. pneumoniae* and no effect against *A. baumannii*. Less evident synergistic activity of **42** was observed with **ERY**, **CAF** and nalidixic acid (**NA**) against the four bacteria. In-depth studies were performed on derivative **41**, as representative, disclosing its ability to inhibit AcrAB–TolC system in EA289 strain (Table 1) by using the fluorescent probe 1,2’-diNA. However, in further experiments the authors also observed a weak disruption of the *S. aureus* and *P. aeruginosa* membranes and an increase in the membrane permeability of EA289, but only at high concentrations. Interestingly, both compounds **41** and **42** showed high CC_50_ values against two different cell lines (L6 rat skeletal myoblast cell and HEK-293 human embryonic kidney cells), thus representing promising EPIs. 

## 4. Nontuberculous Mycobacteria (NTM) Efflux Pump Inhibitors

Nontuberculous mycobacteria (NTM) are microorganisms widely distributed in the environment, mostly present in soil and water, including both natural and treated water sources. 

The NTM species most frequently encountered in clinical practice are: *M. avium*, *M. intracellulare*, *M. kansasii*, *M. xenopi* and *M. abscessus* [97,98]. Some mycobacteria are able to create communities consisting of several species, such as *M. avium complex* (MAC), which includes *M. avium*, *M. intracellulare* and *M. chimaera*.

Unlike the other microorganisms, NTM are not well known and or studied; therefore, few NTM EPIs are reported in literature. 

**Figure 15:**
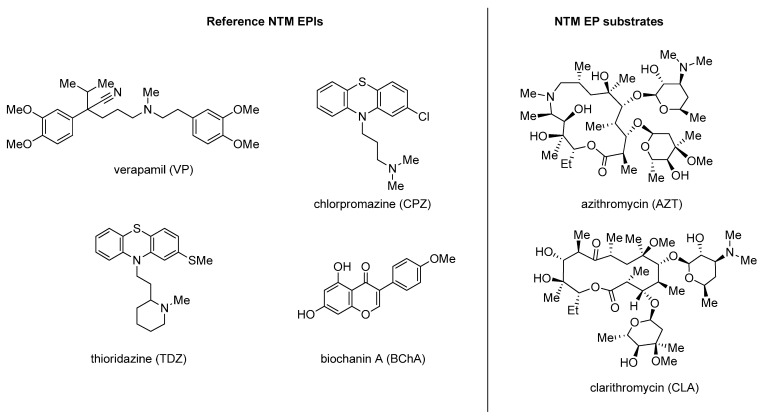
Reference NTM EPIs verapamil (**VP**), chlorpromazine (**CPZ**), thioridazine (**TDZ**) and biochanin A (**BChA**) and NTM EP substrates azithromycin (**AZT**) and clarithromycin (**CLA**).

Only recently, it was shown that NTM EPs are involved in the antimicrobial extrusion and, above all, that their basal expression is very high in these mycobacteria. The classification of EPs in NTM is still in its infancy and, to date, our understanding is based on three recently discovered EPs, MAV_1406 (MFS), MAV_1695 and MAV_3306 (both belonging to ATP-binding cassette) that have been reported to be induced by azithromycin (**AZT**) exposure and related to the appearance of macrolide-resistant phenotypes [99,100]. Indeed, macrolides, such as clarithromycin (**CLA**), **AZT** (Figure 15) and **ERY** (Figure 11), which are first-line drugs in therapy, are substrates of these pumps.

Studies of Rodriguez et al. have shown that NTM resistance to common antimicrobials was significantly reduced by four non-antibiotic drugs with known EPI activity: verapamil (**VP**), chlorpromazine (**CPZ**), thioridazine (**TDZ**) and biochanin A (**BChA**) (Figure 15) [100,101,102,103,104]. 

### Quinoline Derivatives

**Figure 16:**
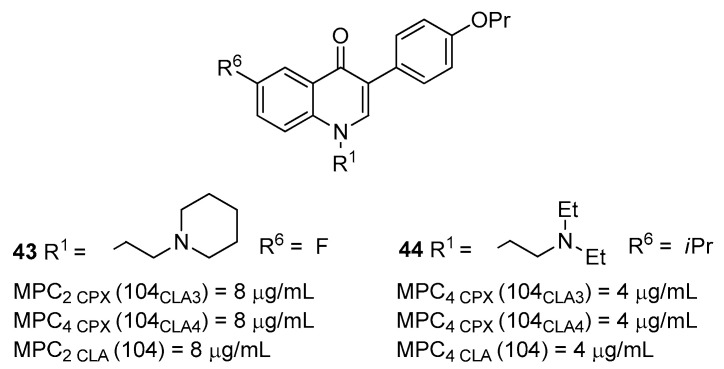
3-phenylquinolones as NTM EPIs.

A set of four quinoline derivatives, previously reported as NorA EPIs, was tested to evaluate their ability to: (i) inhibit **EtBr** efflux, (ii) increase **EtBr** accumulation and (iii) synergize with **CLA** against *M. smegmatis* mc^2^ 155 (ATCC700084—Table 1) [43]. The rational of the initial screening was to identify potential compounds with “broad-spectrum” EPI activity, likely due to the inhibition of NTM EPs belonging to the MFS family as NorA. Compound **23** (Figure 7) exhibited the best EPI activity towards *M. smegmatis* mc^2^ 155, and thus it was advanced for further investigations against several *M. avium* strains. Through synergistic assays, compound **23** was able to synergize with three antibiotics (**CLA**, **ERY** and **AZT**) against different *M. avium* strains with different characteristics, also including the resistance to macrolides due to the overexpression of EPs. As representative, compound **23** at ½ its MIC (ranging from 32 to 64 µg/mL depending on the used *M. avium* strain) was able to reduce antibiotic MICs by values > 512 fold. EP inhibition as mechanism of action of **23** was demonstrated by synergistic assays with **EtBr** against all the used *M. avium* strains and also by **EtBr** efflux and accumulation assays against three different *M. avium* strains differing for the expression of EPs. Unfortunately, **23** exhibited high toxicity towards human monocyte-derived macrophages at the concentrations needed to reach its synergistic effect. Two medicinal chemistry works followed the identification of **23** as NTM EPI, leading to the identification of potent compounds acting at concentrations nontoxic for human monocyte-derived macrophages [105,106]. The initial 2-phenylquinoline structure of **23** was optimized to give 3-phenylquinolone analogues that exhibited a more potent EPI activity synergizing at low concentrations with antibiotics against *M. avium* strains. Therefore, the shift of the phenyl moiety at C-3 position and the functionalization of *N*-1 with alkylamino chains yielding 3-phenylquinolone was beneficial for improving NTM EPI activity, but it was slightly detrimental for NorA EPI activity. Indeed, in a head-to-head comparison between the parent 3-phenylquinolone and 2-phenylquinoline analogues, the former showed a reduced ability to inhibit the **EtBr** efflux on SA-1199B (Table 1) at 50 µM. On the contrary, many 2-phenylquinoline derivatives endowed with potent NorA EPI activity displayed a reduced NTM EPI effect (unpublished data). Taken together, these data suggest a slightly different pharmacophore needed for NorA and NTM EP inhibitions. As a confirmation, the introduction of the OMe group at the C-6 position of 3-phenylquinolone core did not lead to an increase in NTM EPI activity, as previously observed for C-6 OMe 2-phenylquinoline NorA EPIs. On the contrary, at the C-6 position of the 3-phenylquinolone core, a lipophilic moiety was well tolerated, leading to potent and safe NTM EPIs (derivatives **43** and **44**—Figure 16). 

Both compounds showed significant synergism with **CLA**, **CPX** and **EtBr** against three different *M. avium* strains. In particular, **44**, at ^1^/_32_ its MIC (4 µg/mL/8.75 µM), reduced the **CPX** MIC by four-fold against *M. avium* 104_CLA3_ and 104_CLA4_ strains and the **CLA** MIC by four-fold against *M. avium* 104 strain (Table 1). Inhibition of EPs was demonstrated by **EtBr** efflux and accumulation assays against three *M. avium* strains. Due to its CC_50_ of 56.32 µM towards human monocyte-derived macrophages, derivative **44** was also tested at 8.75 µM for its activity against an intracellular infection of *M. avium* 104 in human monocyte-derived macrophages as alone or in combination with **CLA**. The combination of **CLA** and **44** led to a strong boosting of anti-*M. avium* activity with respect to **CLA** alone. Interestingly, although tested at ^1^/_32_ its MIC, **44** also exhibited a similar anti-*M. avium* activity when tested alone, thus suggesting a potential and essential role of the NTM EPs during intracellular infections.

## 5. Conclusions

In this review, we collected all data regarding indole and quinoline derivatives reported in literature as microbial EPIs. When possible, SAR information has been highlighted and discussed to generate a sort of comprehensive SAR around each scaffold with the aim to help the future research of new microbial EPIs.

Regarding the collected information, we observed that most of the compounds (belonging to the quinoline and indole classes) have been reported as NorA EPIs, thus synergizing with the fluoroquinolone **CPX** against different strains of *S. aureus*. Accordingly, the best results in terms of EP inhibition have been obtained against NorA, with some compounds able to synergize with CPX at low concentrations as 0.78 µg/mL [69]. Interestingly, these findings appear in contrast with those observed for EPIs of Gram-negative bacteria. Indeed, although quinoline and indole derivatives showed significant synergism, though often at only high concentrations, the research of Gram-negative EPIs has led to some derivatives reached in vivo studies [86,107]. Accordingly, for AcrAB–TolC (*E. coli*) [108] and MexAB–OprM (*P. aeruginosa*) [109], both EPs belonging to the RND family, there are crystal structures of the whole complexes and, for AcrB and MexB 3D structures in complex with EPIs have been also reported [79,85], thus allowing for future structure-based drug design approaches. 

Therefore, quinoline and indole derivatives would appear to be suitable scaffolds to obtain potent NorA EPIs synergizing with antibiotics at very low concentrations, while representing only tool compounds in the field of Gram-negative EPIs. Of note, it is evident that most of quinoline EPIs need an alkylamino chain to better synergize with antibiotics, in contrast to indole EPIs, where only few derivatives have been reported with basic side chains. Finally, research for NTM EPIs is still in its infancy and, to date, only few molecules have been described in literature. Accordingly, the delineation of a SAR appears complicated, but the few quinoline and quinolone derivatives showing EPI activity can represent a good starting point for future research also aimed at identifying the role of EPs during intracellular infections.

## Figures and Tables

**Table 1:** Description of the cited strains.

Strains	Description	Ref.
***B. subtilis***		
ΔΔ	genes that encode the multidrug transporters Bmr and Blt are genetically inactivated	[26]
ΔΔNA	expresses a functional NorA transporter from the plasmid expression vector pBEV	[27]
***S. aureus***		
SA-1199	wild-type	[27]
SA-1199B	overexpresses the chromosomal *norA* gene which harbors a mutation in *grlA*	[28]
8325-4	wild-type	[29]
K1758	Δ*norA*	[30]
K2361	SA-K1758 with pK364:norA	[31]
ATCC 25923	wild-type	
SA-K2378	having a plasmid that results in overexpression of *norA* from *S. aureus* SA1199	[32]
SA-K1902	*ΔnorA*	[32]
***E. aerogenes***		
ATCC 13048	wild-type	
ATCC 13048 p9	overexpressing the EP activator *marA* gene	[33]
EA27	overexpressing EP, clinically isolated	[34]
EA3	overexpressing EP, clinically isolated	[34]
EA5	overexpressing EP, clinically isolated	[34]
CM-64	resistant to CAF due to EP overexpressing,	[35]
EA117	low porin levels and also substitutions in the QRDR domain of GrlA	[36]
EA289	overexpressing AcrAB–TolC pump	[36]
***E. coli***		
AG100	wild-type	
AG100A	AcrAB pump-deficient	[37]
AG100_tet_	overexpressing efflux pump	[38]
ATCC 25922	wild-type	
YD02	multidrug-resistant after induction from ATCC 25922	[39]
***P. aeruginosa***		
ATCC 27853	wild-type	
***K. pneumoniae***		
KP55	porin deficient phenotype	[40]
***M. smegmatis mc^2^155***		
ATCC 700084	wild-type	[41]
***M. avium***		
104	wild-type	[42]
104_CLA3_	resistant to CLA due to overexpression of efflux pumps	[43]
104_CLA4_	resistant to CLA due to overexpressing efflux pumps and harboring the mutation A-2059G in 23S rRNA	[43]

## Supplementary Materials

The following are available online. Table S1: Chemical name and structure of the described EPI compounds.

## Abbreviations

ABC: ATP-binding cassette; AMR, antimicrobial resistance; ARB, antimicrobial resistance breaker; **AZT**, azithromycin; **BChA**, biochanin A; **CAF**, chloramphenicol; **CEF**, cefepime; **CLA**, clarithromycin; **CPX**, ciprofloxacin; **CPZ**, chlorpromazine; **DOX**, doxycycline; EP, efflux pump; EPI, efflux pump inhibitor; **ERY**, erythromycin; **EtBr**, ethidium bromide; HCT116, human colon tumor; KB, keratin-forming tumor HeLa; HePG2, human liver epithelial; MAC, *M. avium* complex; MATE, multidrug and toxic compound extrusion; MCF7, breast cancer; MCF7R, breast carcinoma; MCR5, human lung fibroblast; MFS, major facilitator superfamily; MIC, minimal inhibitory concentration; MPC, minimal potentiating concentration; MRSA, methicillin resistant *Staphylococcus aureus*; **NFX**, norfloxacin; NTM, nontuberculous mycobacteria; **OFX**, ofloxacin; OM, outer membrane; **OXA**, oxacillin; PACE, proteobacterial antimicrobial compound efflux; **RES**, reserpine; RFF, relative final fluorescence; RND, resistance–nodulation–cell division; SAR, structure-activity relationship; SMR, mall multidrug resistance; **TDZ**, thioridazine; **TET**, tetracycline; THP1, human monocytic cell line; **VP**, verapamil.
