# Supplementary material for: Microbial Efflux Pump Inhibitors: A Journey around Quinoline and Indole Derivatives
Source: Molecules. 2021 Nov 19;26(22):6996. doi: 10.3390/molecules26226996 (PMC8618814; doi:10.3390/molecules26226996)
Supplement: Supplementary file 1 [file molecules-26-06996-s001.zip › molecules-1437775-supplementary.pdf]

**Table S1.** Chemical name and structure of the described EPI compounds

| Compd | Structure | Name |
|---|---|---|
| **1** | 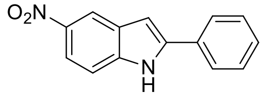 | 5-nitro-2-phenyl-1*H*-indole |
| **2** | 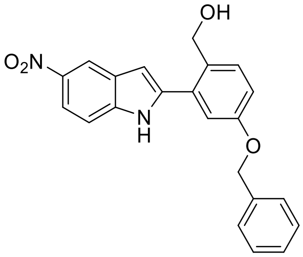 | (4-(benzyloxy)-2-(5-nitro-1*H*-indol-2-yl)phenyl)methanol |
| **3** | 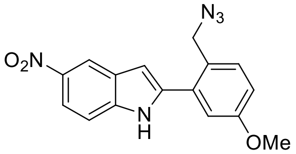 | 2-(2-(azidomethyl)-5-methoxyphenyl)-5-nitro-1*H*-indole |
| **4** | 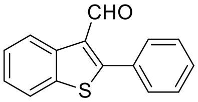 | 2-phenylbenzo[*b*]thiophene-3-carbaldehyde |
| **5** | 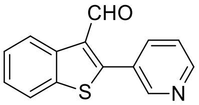 | 2-(pyridin-3-yl)benzo[*b*]thiophene-3-carbaldehyde |
| **6** | 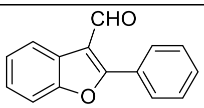 | 2-phenylbenzofuran-3-carbaldehyde |
| **7** | 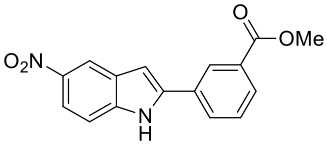 | methyl 3-(5-nitro-1*H*-indol-2-yl)benzoate |
| **8** | 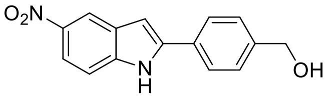 | (4-(5-nitro-1*H*-indol-2-yl)phenyl)methanol |
| **9** | 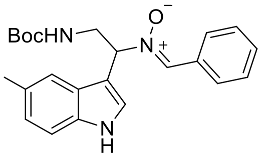 | (Z)-*N*-benzylidene-2-((*tert*-butoxycarbonyl)amino)-1-(5-iodo-1*H*-indol-3-yl)ethanamine oxide |
| **10** | 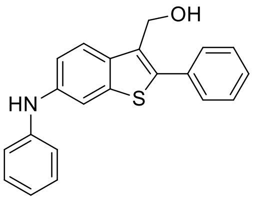 | (2-phenyl-6-(phenylamino)benzo[*b*]thiophen-3-yl)methanol |
| **11** | 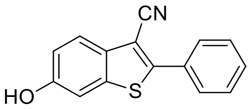 | 6-hydroxy-2-phenylbenzo[*b*]thiophene-3-carbonitrile |

| | | |
|---|---|---|
| **12** | 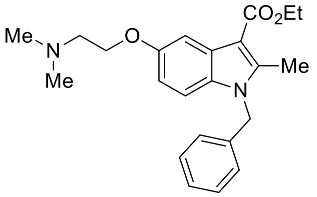 | ethyl 1-benzyl-5-(2-(dimethylamino)ethoxy)-2-methyl-1*H*-indole-3-carboxylate |
| **13** | 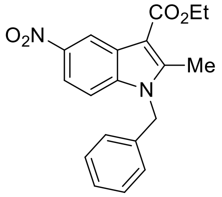 | ethyl 1-benzyl-2-methyl-5-nitro-1*H*-indole-3-carboxylate |
| **14** | 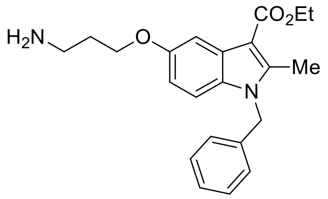 | ethyl 5-(3-aminopropoxy)-1-benzyl-2-methyl-1*H*-indole-3-carboxylate |
| **15** | 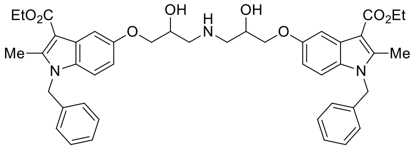 | diethyl 5,5'-((azanediylbis(2-hydroxypropane-3,1-diyl))bis(oxy))bis(1-benzyl-2-methyl-1*H*-indole-3-carboxylate) |
| **16** | 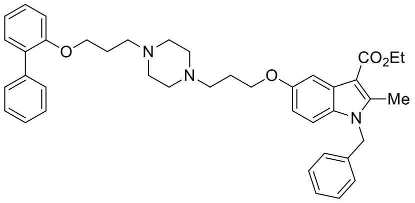 | ethyl 5-(3-(4-(3-([1,1'-biphenyl]-2-yloxy)propyl)piperazin-1-yl)propoxy)-1-benzyl-2-methyl-1*H*-indole-3-carboxylate |
| **17** | 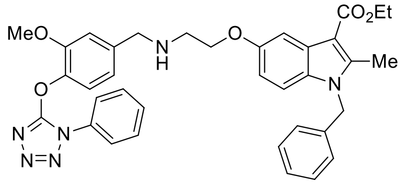 | ethyl 1-benzyl-5-(2-((3-methoxy-4-((1-phenyl-1*H*-tetrazol-5-yl)oxy)benzyl)amino)ethoxy)-2-methyl-1*H*-indole-3-carboxylate |
| **18** | 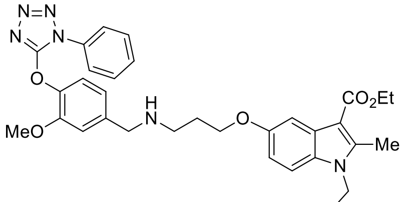 | ethyl 1-benzyl-5-(3-((3-methoxy-4-((1-phenyl-1*H*-tetrazol-5-yl)oxy)benzyl)amino)propoxy)-2-methyl-1*H*-indole-3-carboxylate |
| **19** | 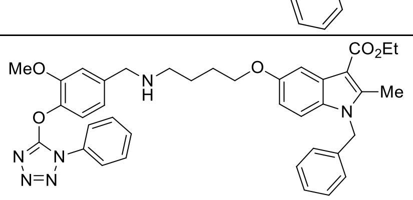 | ethyl 1-benzyl-5-(4-((3-methoxy-4-((1-phenyl-1*H*-tetrazol-5-yl)oxy)benzyl)amino)butoxy)-2-methyl-1*H*-indole-3-carboxylate |

| | | |
|---|---|---|
| **20** | 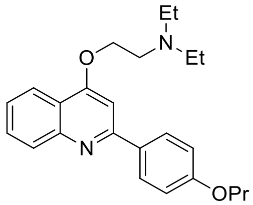 | *N*,*N*-diethyl-2-((2-(4-propoxyphenyl)quinolin-4-yl)oxy)ethanamine |
| **21** | 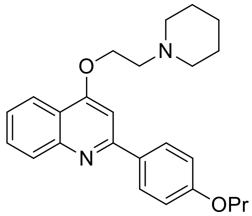 | 4-(2-(piperidin-1-yl)ethoxy)-2-(4-propoxyphenyl)quinoline |
| **22** | 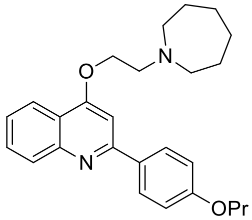 | 4-(2-(azepan-1-yl)ethoxy)-2-(4-propoxyphenyl)quinoline |
| **23** | 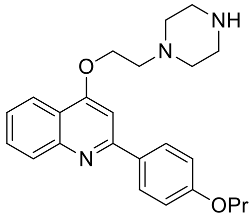 | 4-(2-(piperazin-1-yl)ethoxy)-2-(4-propoxyphenyl)quinoline |
| **24** | 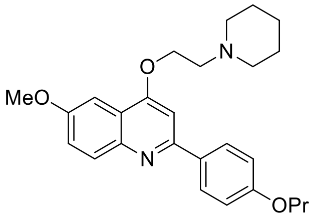 | 6-methoxy-4-(2-(piperidin-1-yl)ethoxy)-2-(4-propoxyphenyl)quinoline |
| **25** | 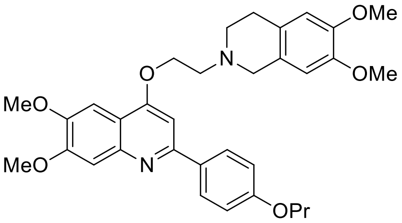 | 4-(2-(6,7-dimethoxy-3,4-dihydroisoquinolin-2(1*H*)-yl)ethoxy)-6,7-dimethoxy-2-(4-propoxyphenyl)quinoline |
| **26** | 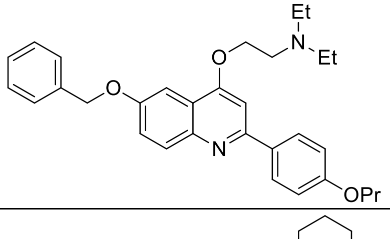 | 2-((6-(benzyloxy)-2-(4-propoxyphenyl)quinolin-4-yl)oxy)-*N*,*N*-diethylethanamine |
| **27** | 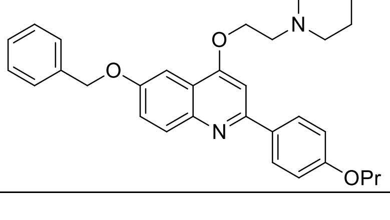 | 6-(benzyloxy)-4-(2-(piperidin-1-yl)ethoxy)-2-(4-propoxyphenyl)quinoline |

| | | |
|---|---|---|
| **28** | 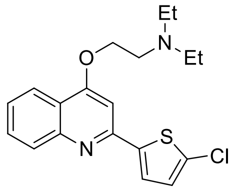 | 2-((2-(5-chlorothiophen-2-yl)quinolin-4-yl)oxy)-*N*,*N*-diethylethanamine |
| **29** | 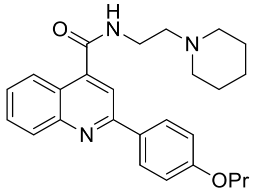 | *N*-(2-(piperidin-1-yl)ethyl)-2-(4-propoxyphenyl)quinoline-4-carboxamide |
| **30** | 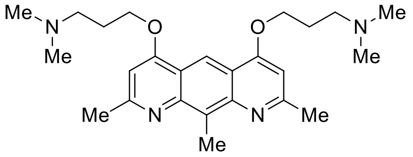 | 3,3'-((2,8,10-trimethylpyrido[3,2-*g*]quinoline-4,6-diyl)bis(oxy))bis(*N*,*N*-dimethylpropan-1-amine) |
| **31** | 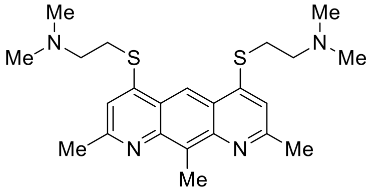 | 2,2'-((2,8,10-trimethylpyrido[3,2-*g*]quinoline-4,6-diyl)bis(sulfanediyl))bis(*N*,*N*-dimethylethanamine) |
| **32** | 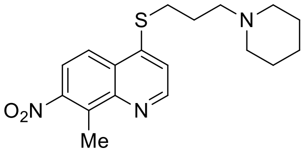 | 8-methyl-7-nitro-4-((3-(piperidin-1-yl)propyl)thio)quinoline |
| **33** | 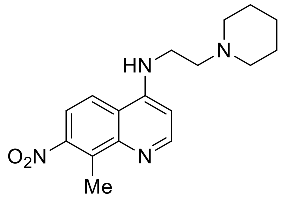 | 8-methyl-7-nitro-*N*-(2-(piperidin-1-yl)ethyl)quinolin-4-amine |
| **34** | 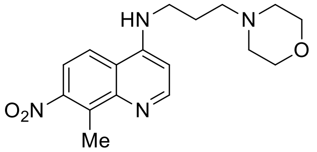 | 8-methyl-*N*-(3-morpholinopropyl)-7-nitroquinolin-4-amine |
| **35** | 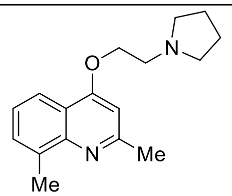 | 2,8-dimethyl-4-(2-(pyrrolidin-1-yl)ethoxy)quinoline |
| **36** | 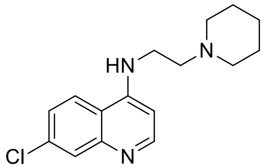 | 7-chloro-*N*-(2-(piperidin-1-yl)ethyl)quinolin-4-amine |

| | | |
|---|---|---|
| **37** | 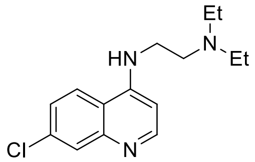 | $N^1$-(7-chloroquinolin-4-yl)-$N^2$,$N^2$-diethylethane-1,2-diamine |
| **38** | 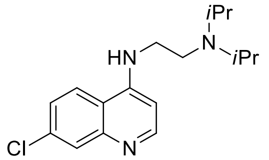 | $N^1$-(7-chloroquinolin-4-yl)-$N^2$,$N^2$-diisopropylethane-1,2-diamine |
| **39** | 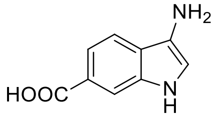 | 3-amino-1*H*-indole-6-carboxylic acid |
| **40** | 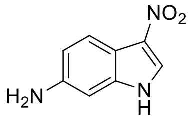 | 3-nitro-1*H*-indol-6-amine |
| **41** | 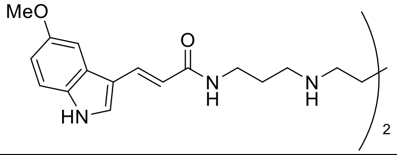 | (2*E*,2'*E*)-*N*,*N*'-((butane-1,4-diylbis(azanediyl))bis(propane-3,1-diyl))bis(3-(5-methoxy-1*H*-indol-3-yl)acrylamide) |
| **42** | 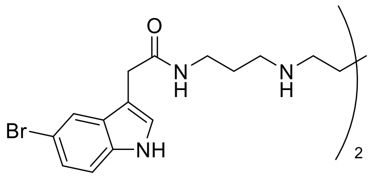 | *N*,*N*'-((pentane-1,5-diylbis(azanediyl))bis(propane-3,1-diyl))bis(2-(5-bromo-1*H*-indol-3-yl)acetamide) |
| **43** | 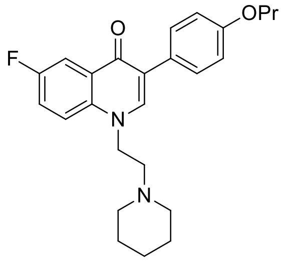 | 6-fluoro-1-(2-(piperidin-1-yl)ethyl)-3-(4-propoxyphenyl)quinolin-4(1*H*)-one |
| **44** | 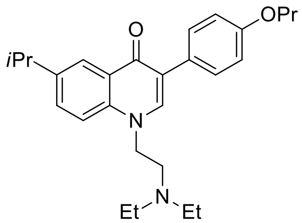 | 1-(2-(diethylamino)ethyl)-6-isopropyl-3-(4-propoxyphenyl)quinolin-4(1*H*)-one |
